# Overexpression of long noncoding RNA colorectal neoplasia differentially expressed protects spinal cords against ischemia by targeting microRNA-181a-5p/Sirtuin-1 axis

**DOI:** 10.3389/fneur.2026.1825718

**Published:** 2026-05-12

**Authors:** Zhi Wang, Wei Wang, Rui Tang, Shilun Gao, Yubao Zhu, Tianxiang Gu, Xiaojing Jiang, Enyi Shi

**Affiliations:** 1 Department of Cardiac Surgery, First Affiliated Hospital, China Medical University, Shenyang, China; 2 Department of Cardiac Surgery, The People’s Hospital of Bozhou, Anhui, China; 3 State Key Laboratory of Cardiovascular Disease, Center of Vascular Surgery, Fuwai Hospital, National Center for Cardiovascular Diseases, Chinese Academy of Medical Sciences and Peking Union Medical College, Beijing, China; 4 Department of Anesthesiology, First Affiliated Hospital, China Medical University, Shenyang, China

**Keywords:** ischemia–reperfusion, lncRNA CRNDE, microRNA-181a-5p, SIRT 1, spinal cord

## Abstract

**Objective:**

The objective of this study was to investigate the neuroprotective effects of long non-coding RNA colorectal neoplasia differentially expressed (CRNDE) on ischemic spinal cords.

**Materials and methods:**

The binding relationship between CRNDE and microRNA-181a-5p was detected using dual luciferase assays. Spinal cord ischemia was induced in rats by cross clamping the descending aorta. CRNDE expression was induced by intrathecal injection of adeno-associated virus vectors containing CRNDE. The hind-limb motor function of the rats was then assessed over a period of 3 weeks following reperfusion. Lumbar spinal cords were harvested for histologic examinations. Expressions of CRNDE, microRNA-181a-5p and related proteins were measured by quantitative reverse transcription polymerase chain reaction and Western blot.

**Results:**

Luciferase assays demonstrated that CRNDE bound to microRNA-181a-5p, and Sirt1 was a direct target of microRNA-181a-5p. The transient ischemia induced a significant decrease of CRNDE expression accompanied by a robust increase of microRNA-181a-5p expression in spinal cords. Intrathecal injection of adeno-associated virus vectors containing CRNDE resulted in a significant enhancement of CRNDE expression and a repression of microRNA-181a-5p expression in spinal cords. Consequently, CRNDE overexpression was found to inhibit neuronal apoptosis, attenuate histologic damage, increase the number of surviving neurons, and improve the hind-limb motor function after spinal cord ischemia.

**Conclusion:**

CRNDE overexpression induces spinal cord protection against ischemia–reperfusion injury, possibly via microRNA-181a-5p/Sirt1 axis.

## Graphical abstract

**Figure:**
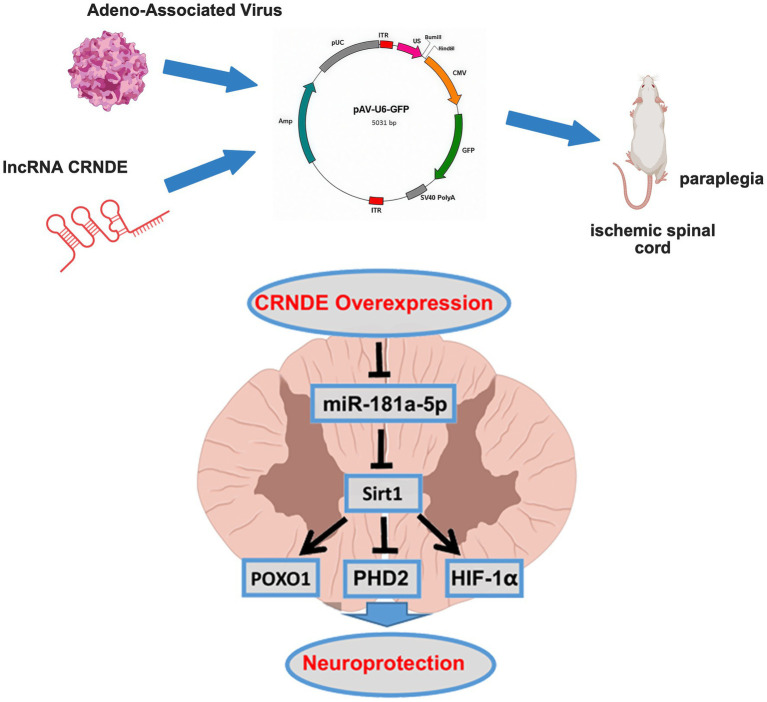
CRNDE overexpression induces protection against spinal cord ischemia, possibly by direct binding to miR-181a-5p and upregulation of Sirt1. The downstream targets of Sirt1, including FOXO1, HIF-1α, and PHD2, may be also involved in the neuroprotection.

## 1 Introduction

Despite the continuous improvement in the mortality and morbidity rates of open and endovascular thoracoabdominal aortic aneurysm repair, attributable to the development of surgical adjuncts and pharmacologic interventions, paraplegia or paralysis secondary to transient or permanent spinal cord ischemia remains a prevalent and grave complication (1).

Long non-coding RNA (lncRNA) is a class of non-coding RNA that is more than 200 nucleotides in length and does not possess protein-coding ability. LncRNAs have been demonstrated to exert a profound influence on a multitude of cellular processes, including the development and injury of the central nervous system (2, 3). Furthermore, the ability of these molecules to function as competing endogenous RNAs (ceRNAs) by binding to microRNAs (miRs) and thereby reducing their regulatory effects on messenger RNA has been demonstrated. Mounting evidence indicates that these molecules can regulate key factors involved in ischemic stroke, such as calcium overload, glutamate toxicity, autophagy, inflammation, and oxidative stress (4). LncRNA metastasis-associated lung adenocarcinoma transcript 1 (MALAT1) has been shown to induce anti-apoptotic and anti-inflammatory effects on brain microvasculature, thereby reducing ischemic cerebral vascular and parenchymal damages (5). Furthermore, the study demonstrates that the upregulation of H19, a non-coding RNA, has been shown to promote neuroinflammation by driving histone deacetylase 1-dependent M1 microglial polarization, and that the downregulation of H19 has been demonstrated to reduce infarct volume and brain edema (6). Furthermore, the inhibition of LncRNA growth arrest-specific 5 has been shown to provide neuroprotection against ischemic cerebral injury induced by deep hypothermia circulatory arrest (7). However, the role and underlying mechanisms of these non-coding RNA molecules in spinal cord ischemia remain to be fully elucidated.

As a lncRNA initially identified in colorectal cancer (8, 9), colorectal neoplasia differentially expressed (CRNDE) has been shown to exert oncogenic functions in a range of cancers, including gliomas (10). CRNDE modulates mammalian target of rapamycin signaling in glioma and promotes cell growth and migration *in vitro* and in a xenograft mouse model (10, 11). The promotion of malignant progression of glioma by CRNDE is achieved through the miR-23b-3p/isocitrate dehydrogenase 1 (IDH1) axis (11). Furthermore, the study observed that CRNDE displays tissue-specific expression patterns, with the highest levels of expression being detected in the deep central structures of the brain and spinal cords (12). It is further postulated that CRNDE may be implicated in lineage specification in the mammalian brain and act as a significant contributor to neurodegenerative processes (12). Collective evidence also indicates that CRNDE participates in inflammation development, progression of sepsis and the pathological process of ischemic injury (13, 14).

Although numerous ceRNA networks have been shown to confer neuroprotection in cerebral ischemia–reperfusion injury, their roles in spinal cord ischemia–reperfusion injury remain largely underexplored. Here, we test the hypothesis that CRNDE, a CNS-enriched lncRNA (12), acts as a ceRNA to exert neuroprotective effects following transient spinal cord ischemia–reperfusion injury.

## 2 Materials and methods

### 2.1 Animals

Male Wistar rats (10 weeks old, approximately 250 g) were obtained from Liaoning Changsheng Biological Technology Co., Ltd. (Liaoning, China) and housed under standard laboratory conditions with a 12-h light/dark cycle and free access to food and water. The experimental protocol was approved by the Institutional Animal Care and Use Committee (IACUC) at our institution, in accordance with the ARRIVE guidelines for reporting *in vivo* experiments (15).

### 2.2 Luciferase assays

CRNDE full-length and Sirt1 3′-UTR sequences were synthesized and inserted into a pmirGlo Dual-luciferase miR Target Expression Vector (CRNDE-WT and Sirt1-WT), with the aim of constructing a luciferase reporter vector. Subsequently, the sequences were replaced to mutate the putative binding sites (CRNDE-MT and Sirt1-MT). Subsequently, HEK-293 T cells were cotransfected with CRNDE-WT (or CRNDE-MT), Sirt1-WT (or Sirt1-MT) and miR-181a-5p mimics or mimics NC plasmids. Forty-eight hours post-transfection, the luciferase activities were measured through a dual luciferase reporter assay system according to the manufacturer’s protocol (Beijing Syngenbio Co., Beijing, China).

### 2.3 Upregulation of CRNDE *in vivo*

The full-length CRNDE-rat sequence (gene ID: 106456572) was synthesized and cloned into Adeno-associated virus (AAV) vectors (AAV-LncRNA CRNDE-GFP) (Beijing Syngenbio Co, Beijing, China). AAV vectors devoid of the LncRNA CRNDE (AAV-GFP) were utilized as controls. The AAV-LncRNA CRNDE-GFP or AAV-GFP vectors were injected into the spinal cords of subjects via the intrathecal route, as previously outlined (16).

### 2.4 Spinal cord ischemia

As reported previously, the induction of spinal cord ischemia was achieved through the implementation of cross-clamping of the descending aorta, occurring just distal to the left subclavian artery, for a period of 15 min (16).

### 2.5 Experimental protocol

In our experimental procedures, all animals were euthanized using an overdose of pentobarbital sodium (150 mg/kg, intraperitoneal injection), in accordance with institutional guidelines and ethical standards for animal welfare. This method ensures rapid loss of consciousness followed by death without pain or distress. Animals were randomly assigned to their respective groups using a computer-generated random number table. In order to ascertain the time course for expressions of CRNDE and miR-181a-5p, rats were subjected to a 15-min spinal cord ischemia and then killed at 6 h, 12 h, 24 h, 1 week and 3 weeks after reperfusion (*n* = 4, at each time point). An additional four rats underwent a surgical procedure without spinal cord ischemia and served as a control group.

The neuroprotective effect of CRNDE overexpression was evaluated in four groups: (1) Sham group: rats underwent only the surgical procedure without ischemia; (2) Control group: rats underwent spinal cord ischemia without any further intervention; (3) Vector group: rats underwent spinal cord ischemia and received an intrathecal injection of AAV-GFP; and (4) LncRNA CRNDE group: rats underwent spinal cord ischemia and received an intrathecal injection of AAV-LncRNA CRNDE-GFP. Injections were performed 10 days before the ischemia procedure. To ensure unbiased assessment, researchers performing the behavioral and histological evaluations were blinded to the group assignments.

Concurrently, a series of experiments was conducted in which spinal cords were collected 12 h after reperfusion from the four groups to evaluate expressions of CRNDE, miR-181-5p, Sirt1, Forkhead box O1 (FOXO1), Hypoxia inducible factor (HIF)-1α and Prolyl hydroxylase 2 (PHD2). Subsequent to this, rats from the four groups were euthanised 24 h after reperfusion in order to assess neuronal apoptosis.

### 2.6 Neurologic assessment

The assessment of hind-limb motor function was conducted at 24 h, 48 h, 72 h, 1 week, and 3 weeks following reperfusion. The Motor Deficit Index (MDI) score was utilized to quantify motor function, with the ambulation and placing/stepping reflex being the primary metrics. Investigators who were unaware of group allocation information performed this assessment (16).

### 2.7 Immunohistochemistry

For the purpose of immunohistochemistry, spinal cords harvested a period of 3 weeks following reperfusion were frozen and sectioned. NeuN (Invitrogen, Carlsbad, CA) was used to label neurons and 4′,6-diamidino-2-phenylindole (DAPI) (Invitrogen, Carlsbad, CA) was used as a nuclear counterstain. The NeuN positive (red fluorescent) cells in the ventral part of the gray matter were counted in three individual sections by an assessor who was blind to group information.

### 2.8 Western blot

The expression levels of Sirt1, FOXO1, HIF-1α and PHD2 in the spinal cords of the subjects were analyzed by sodium dodecyl sulfate polyacrylamide gel electrophoresis and immunoblotted with anti-Sirt1, anti-FOXO1, anti-HIF-1 and anti-cleaved PHD2 antibodies (Abcam, Cambridge, Mass). The protein expressions were analyzed with the National Institutes of Health Image (Research Services Branch, National Institutes of Health, Bethesda, Md) and quantified as a relative fold to the sham group after normalization with glyceraldehyde-3-phosphate dehydrogenase (GAPDH).

### 2.9 Quantitative real-time polymerase chain reaction

Total RNA was isolated by means of the Trizol reagent (Invitrogen, Carlsbad, CA). Quantitative real-time polymerase chain reaction (PCR) was then performed according to a standard protocol using the StepOne Plus system (Applied Biosystems, Foster City, CA). U6 served as an internal control. Changes in expression were determined by the 2^−*∆∆CT*^ method.

### 2.10 TUNEL staining

Frozen sections were utilized for *in situ* TUNEL staining, employing a one-step TUNEL labeling kit (Beyotime, Shanghai, China). Fluorescence signals were then visualized via the utilization of a fluorescence microscope, and the ratio of positive neurons was subsequently calculated in the ventral part of the gray matter.

### 2.11 Histologic study

For the purpose of histologic study, paraffin-embedded sections of lumbar spinal cords (L4–L6) were subjected to staining with Nissl dye.

### 2.12 Statistical analysis

The determination of the sample size was principally based on power analysis of pilot data with a power of 0.80 and *α* = 0.05. The values of CRNDE, miR-181a-5p, Sirt1, FOXO1, HIF-1α, PHD2 and the apoptosis rate were reported as the mean ± standard deviation (SD) and analyzed by one-way analysis of variance (ANOVA) followed by Bonferroni correction. MDI scores and numbers of neurons were analyzed using the Kruskal-Wallis test with Bonferroni correction (*p* < 0.008 was considered statistically significant). The statistical analysis was performed with SPSS, version 19.0 (IBM-SPSS Inc., Armonk, NY).

## 3 Results

### 3.1 LncRNA CRNDE downregulation was associated with miR-181a-5p upregulation in spinal cords after the transient ischemia

**Figure 1:**
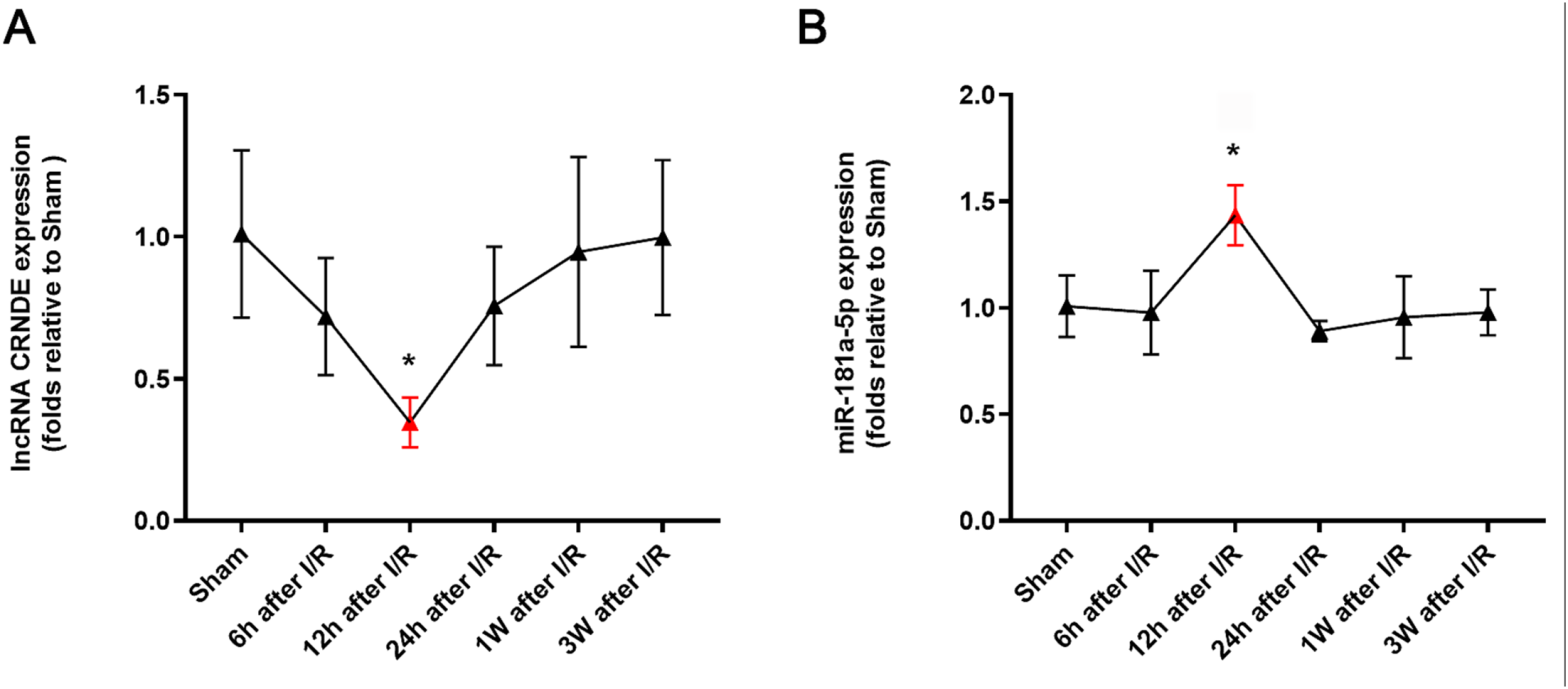
Time course of expressions of CRNDE **(A)** and miR-181a-5p **(B)** in spinal cords after transient ischemia. *n* = 4, each group. *p < 0.05 versus Sham.

CRNDE expressions in the spinal cords of subjects in the study group were downregulated, reaching a nadir at 12 h after reperfusion (*p* = 0.02 vs. the Sham) (Figure 1A). Following this, CRNDE expressions increased, and no significant differences were detected at 24 h, 1 week and 3 weeks after reperfusion when compared with the Sham group (*p* > 0.05 vs. the Sham, respectively). Concurrent with the downregulation of CRNDE at 12 h following reperfusion, there was a significant upregulation of miR-181a-5p expression in spinal cords (*p* = 0.01 vs. the Sham) (Figure 1B).

### 3.2 CRNDE targeted and repressed miR-181a-5p expression

**Figure 2:**
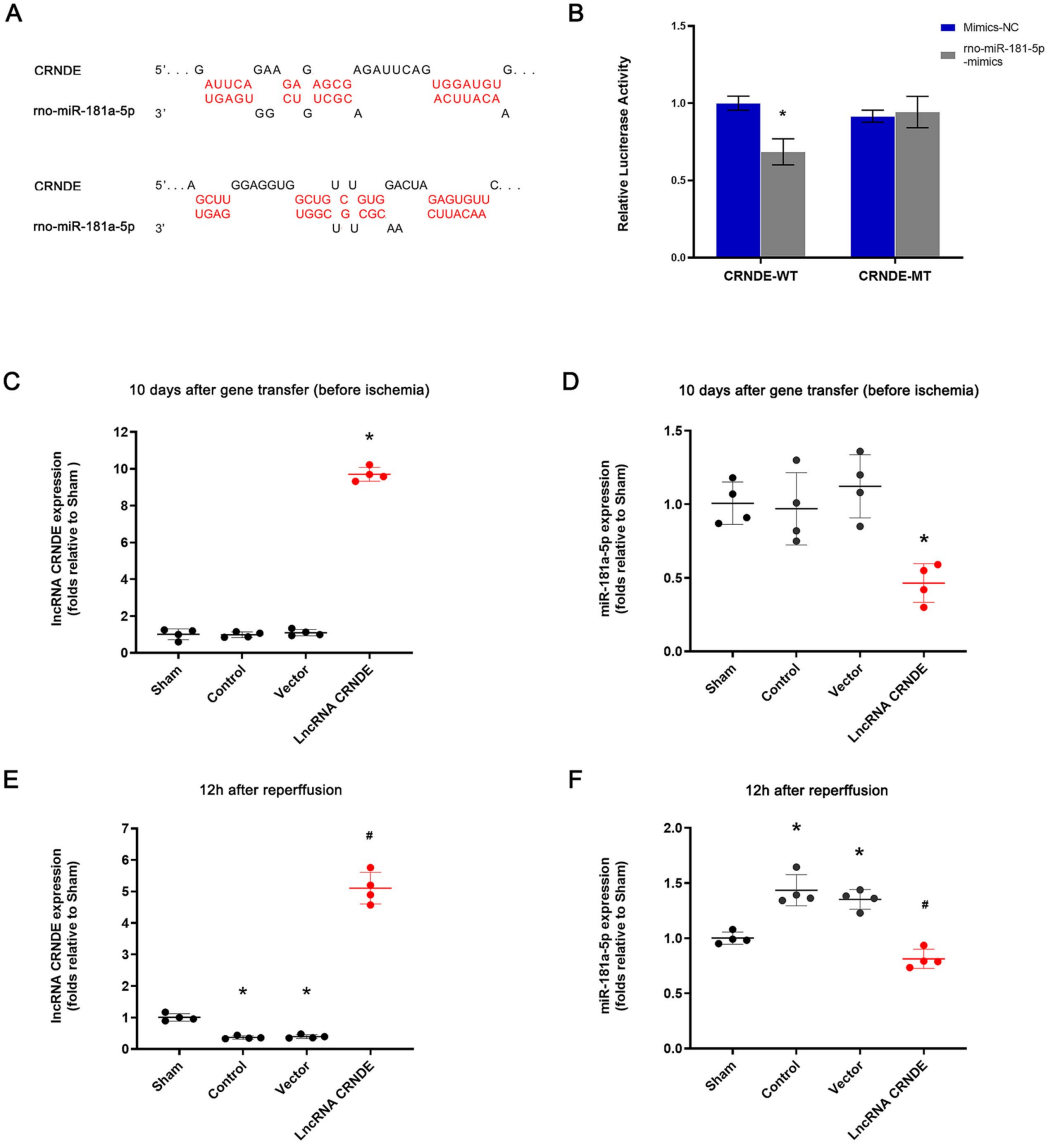
CRNDE bound to miR-181a-5p and repressed its expression. **(A)** Schematic illustration of the 2 predicted binding sites between CRNDE and miR-181a-5p. **(B)** The relative luciferase activities were inhibited in the HEK-293 T cells co-transfected with the reporter vector CRNDE wild-type and miR-181a-5p mimic. *p < 0.001 versus co-transfer of CRNDE wild-type and miR-181a-5p mimic-NC. **(C)** Expression of CRNDE in spinal cords was significantly enhanced 10 days after gene transfer of lncRNA CRNDE. *n* = 4, each group. *p < 0.001 versus Sham. **(D)** Expression of miR-181a-5p in spinal cords was significantly inhibited 10 days after gene transfer of lncRNA CRNDE. *n* = 4, each group. *p = 0.01 versus Sham. **(E)** Expression of CRNDE in spinal cords was significantly enhanced by gene transfer of lncRNA CRNDE 12 h after transient ischemia. *n* = 4, each group. *p < 0.001 versus Sham. ^#^*p* < 0.001 versus Control. **(F)** Expression of miR-181a-5p in spinal cords was significantly inhibited by gene transfer of lncRNA CRNDE 12 h after transient ischemia. *n* = 4, each group. *p < 0.01 versus Sham. ^#^*p* < 0.001 versus Control.

Potential binding sites between CRNDE and miR-181a-5p were predicted using StarBase v3.0/ENCORI (17) and TargetScan Human 8.0 (18) (Figure 2A). In order to ascertain the binding sites of CRNDE and miR-181a-5p, dual-luciferase gene reporter assays were performed. The relative luciferase activities of cotransfection of CRNDE WT and miR-181a-5p mimics were significantly attenuated in HEK-293 T cells (*p* < 0.001) (Figure 2B), whereas the luciferase activity in the CRNDE MT groups was not affected.

Furthermore, CRNDE expression in the spinal cords of subjects was enhanced significantly 10 days after intrathecal injection of AAV vectors containing CRNDE (*p* < 0.001 vs. the Sham group) (Figure 2C). Concomitantly, the expression of microRNA-181a-5p was significantly repressed by transfer of CRNDE (*p* = 0.01 vs. the Sham group) (Figure 2D). Furthermore, 12 h following spinal cord ischemia, a significant decrease in CRNDE expression was observed in both the Control and Vector groups (*p* = 0.005 and *p* = 0.006, respectively, vs. the Sham group). Conversely, CRNDE expression in the LncRNA CRNDE group was considerably higher than that in the Control group (*p* < 0.001, Figure 2E). Furthermore, transfer of CRNDE markedly attenuated miR-181a-5p expression at 12 h after reperfusion (the LncRNA CRNDE group vs. the Control group, *p* < 0.001) (Figure 2F).

### 3.3 CRNDE overexpression activated Sirt1 pathway in spinal cords by sponging miR-181a-5p

**Figure 3:**
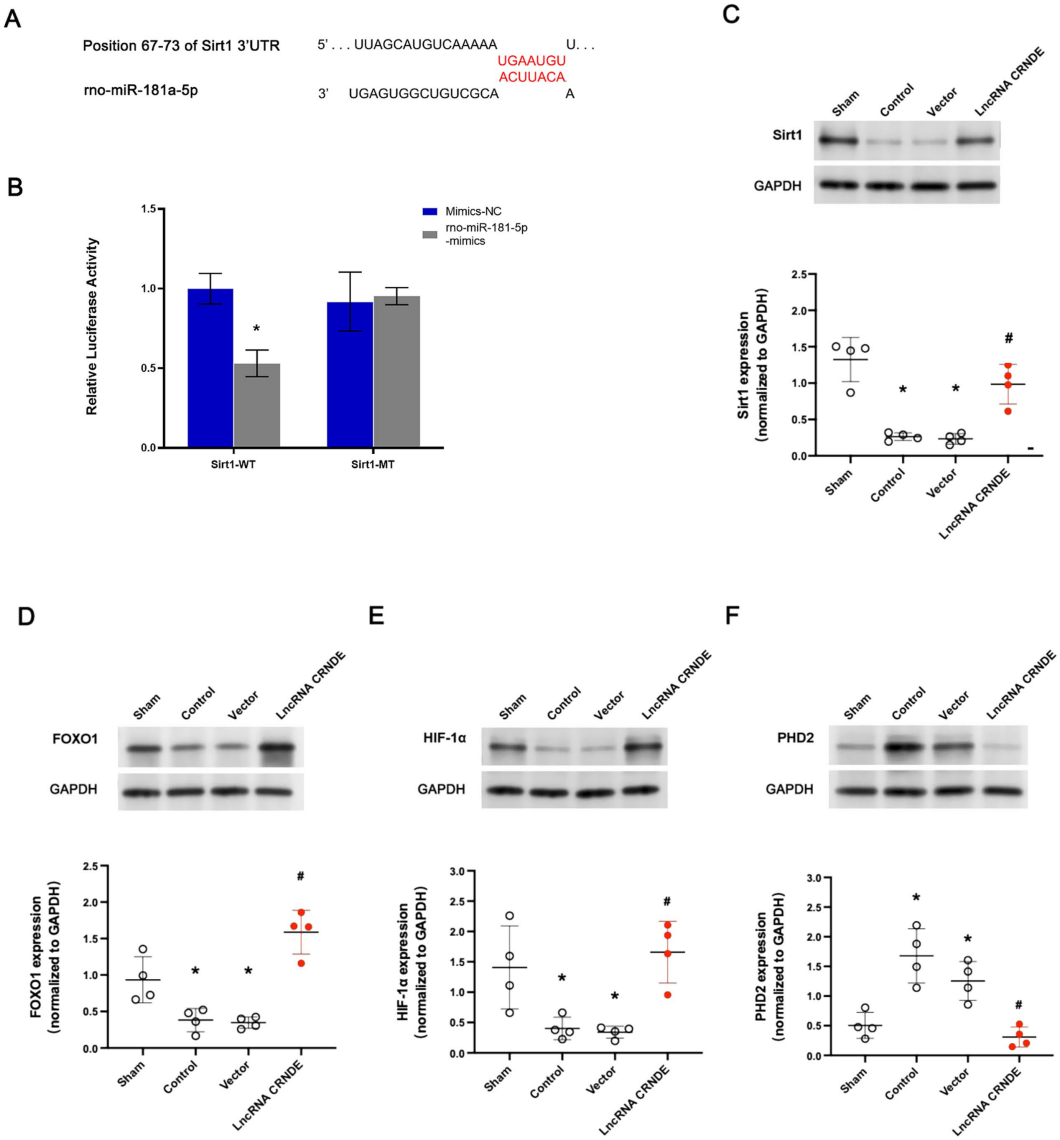
CRNDE overexpression regulated Sirt1 pathway in spinal cords by sponging miR-181a-5p. **(A)** Schematic illustration of the predicted binding site between miR-181a-5p and Sirt1. **(B)** miR-181a-5p significantly inhibited the luciferase activity of Sirt1 3′UTR wild type. *p < 0.001 versus co-transfer of Sirt1 3′UTR wild type and miR-181a-5p mimic-NC. **(C)** Expression of Sirt1 was significantly repressed in spinal cords after transient ischemia, which was dramatically enhanced by gene transfer of lncRNA CRNDE. *n* = 4, each group. *p < 0.001 versus Sham. ^#^*p* = 0.01 versus Control. **(D)** Spinal cords FOXO1 expression after transient ischemia was markedly increased by gene transfer of lncRNA CRNDE. *p < 0.05 versus Sham. ^#^*p* = 0.001 versus Control. **(E)** Spinal cords HIF-1α expression after ischemia was dramatically upregulated by gene transfer of lncRNA CRNDE. *p < 0.05 versus Sham. ^#^*p* = 0.002 versus Control. **(F)** Spinal cords PHD2 expression after transient ischemia was remarkably decreased by gene transfer of lncRNA CRNDE. *p < 0.05 versus Sham. **p < 0.01 versus Sham. ^#^*p* = 0.001 versus Control.

Since Sirt1 is known to mediate neuroprotection primarily through deacetylation of FOXO1 and regulation of the HIF-1α/PHD2 axis in other ischemic models, we next examined these key downstream effectors in spinal cord tissue. Consistent with above predictions, Sirt1 was identified as a direct target of miR-181a-5p, with conserved binding sites in its 3′-UTR (Figure 3A). The results demonstrate a significant repression of luciferase activity in the Sirt1 WT + miR-181a-5p mimics group in comparison with the Sirt1 WT + miR-181a-5p mimics NC group (*p* < 0.001). However, the luciferase activity between the Sirt1 MT + miR-181a-5p mimics and Sirt1 MT + miR-181a-5p mimics NC groups showed no significant difference (*p* = 1.000) (Figure 3B).

The transient ischemia induced significant decreases of Sirt1 expression in the Control and the Vector groups (*p* < 0.001 and *p* < 0.001, vs. the Sham group, respectively) (Figure 3C). Conversely, the LncRNA CRNDE group exhibited a marked increase in Sirt1 expression (*p* = 0.010, vs. the Control group).

In comparison with the Sham group, the Control group (FOXO1, *p* = 0.027; HIF-1α, *p* = 0.021) and the Vector group (FOXO1, *p* = 0.015; HIF-1α, *p* = 0.016) exhibited significantly diminished expressions of FOXO1 (Figure 3D) and HIF-1α (Figure 3E) in the spinal cords following spinal cord ischemia. The present study set out to investigate the impact of transfection of CRNDE on the expression of FOXO1 and HIF-1α. The results obtained demonstrated a significant enhancement in the decrease of expressions of both FOXO1 and HIF-1α in the LncRNA CRNDE group (FOXO1, *p* < 0.001, vs. the Control group; HIF-1α, *p* < 0.001, vs. the Control group). The PHD2 expression (Figure 3F) in the spinal cords increased significantly after transient ischemia in the Control (*p* = 0.002, vs. the Sham group) and Vector groups (*p* = 0.048, vs. the Sham group). However, transfection of CRNDE significantly reduced the expression of PHD2 in the LncRNA CRNDE group (*p* = 0.001, vs. the Control group).

### 3.4 CRNDE overexpression improved hind-limb motor function after spinal cord ischemia

The protocol for assessing hind limb motor function for 3 weeks after spinal cord ischemia included 39 rats. Seven rats were excluded from the study due to death during the surgical procedure or due to the fact that they did not survive for 3 weeks after reperfusion. Complete data were obtained for the remaining 32 rats (*n* = 8 per group).

**Figure 4:**
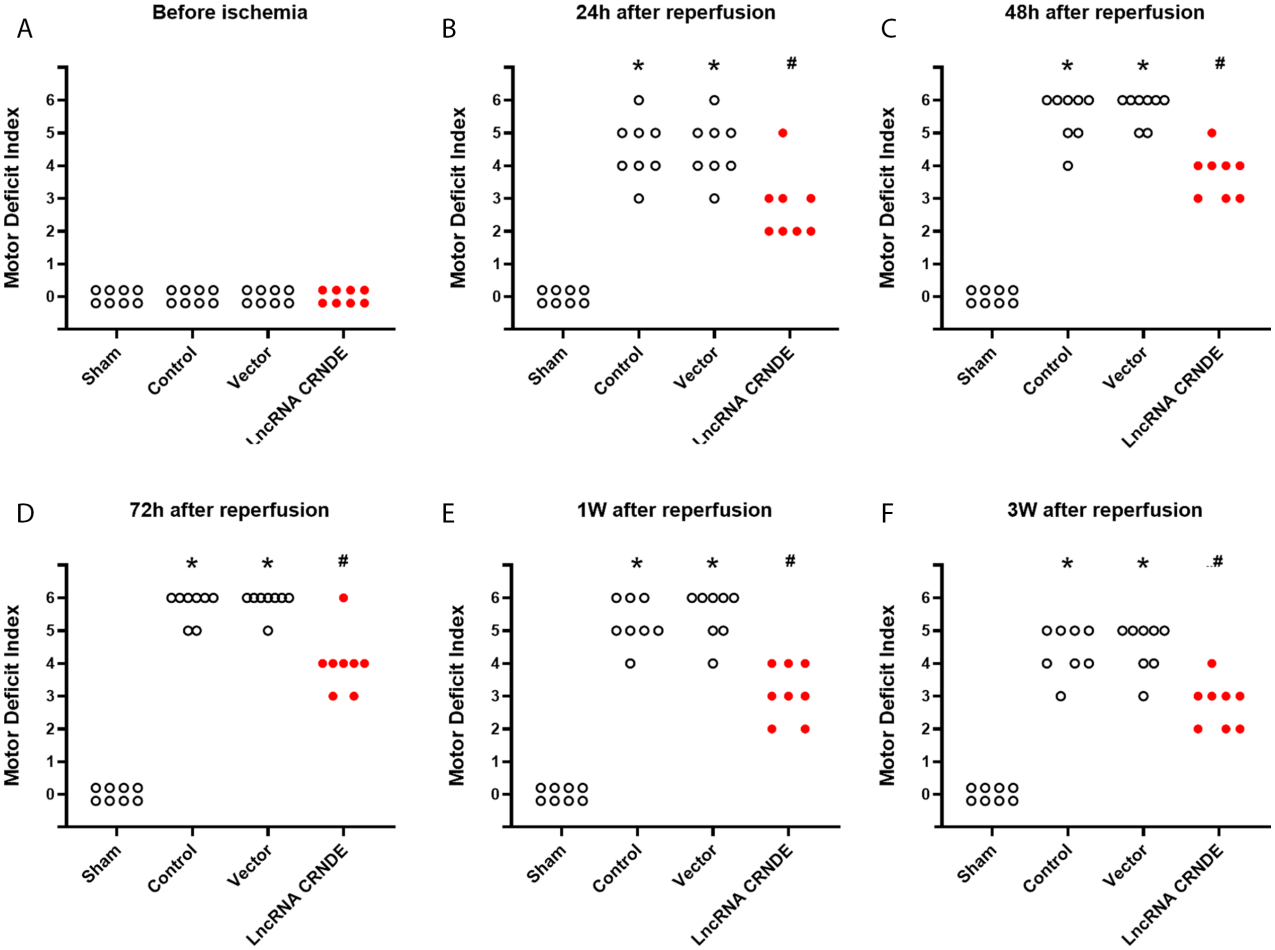
Effects of upregulation of CRNDE on hind-limb motor function assessed with motor deficit index before spinal cords ischemia **(A)**, and 24 h **(B)**, 48 h **(C)**, 72 h **(D)**, 1 week **(E)** and 3 weeks **(F)** after reperfusion. *n* = 8, each group. *p < 0.001 versus Sham. ^#^*p* < 0.008 versus Control.

Intrathecal injection did not affect hind-limb motor function, and all rats in the Control, Vector and LncRNA CRNDE groups demonstrated normal MDI scores prior to the induction of spinal cord ischemia (Figure 4). However, severe hind-limb neurological deficits were induced by the 15-min spinal cord ischemia in both the Control and Vector groups (*p* < 0.001 vs. the sham group at the 5 time points after reperfusion, respectively). In comparison with the Control group, the MDI scores of the LncRNA CRNDE group were significantly lower at the five observation time points following reperfusion (*p* = 0.007, 0.002, 0.003, 0.001, and 0.003, respectively).

### 3.5 CRNDE overexpression inhibited neuronal apoptosis induced by spinal cord ischemia

**Figure 5:**
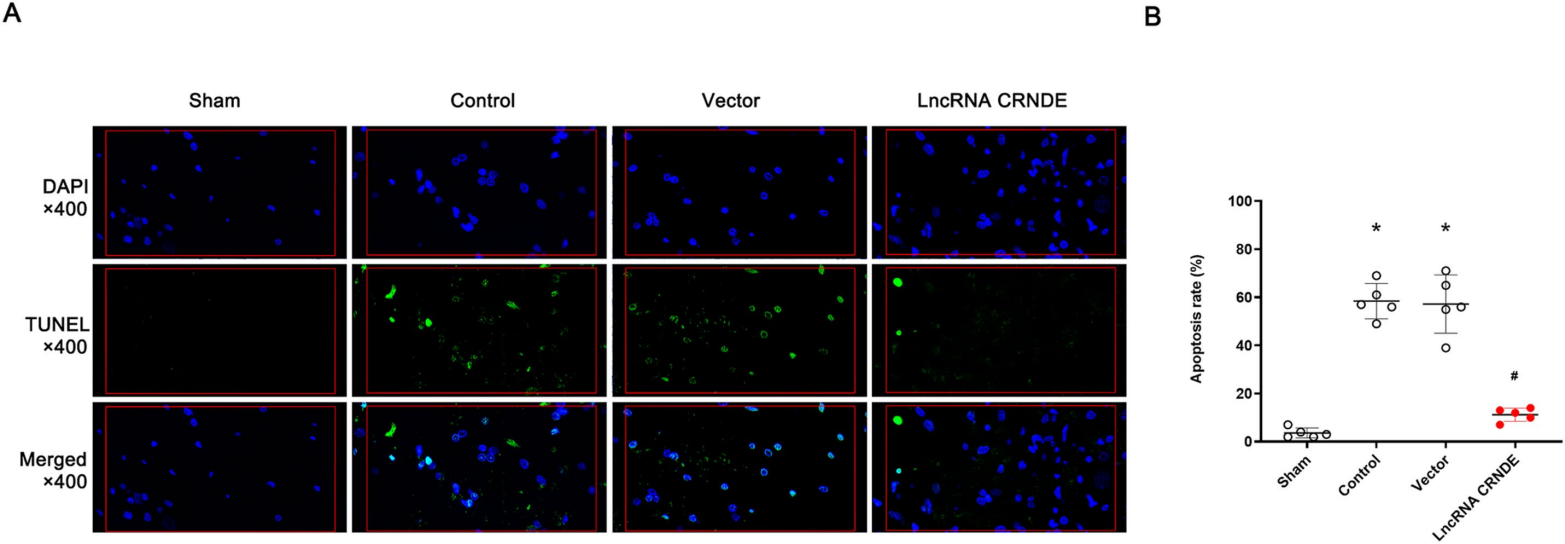
Overexpression of CRNDE inhibited neuronal apoptosis induced by transient spinal cords ischemia. **(A)** Immunofluorescent images of TUNEL staining (green) sections double staining with 4′6-diamidino-2-phenylindole (DAPI) (blue). **(B)** Ratio of apoptotic neurons in the ventral gray matter of lumber spinal cords. *p < 0.001 versus Sham. ^#^*p* < 0.001 versus Control.

As illustrated by the immunofluorescent images of TUNEL staining in Figure 5A, numerous TUNEL-positive cells were observed in both the Control and Vector groups, whereas almost no apoptotic signals were detected in the Sham group, confirming the success of the spinal cord ischemia model. Quantitative analysis in Figure 5B showed that the apoptosis rates in the Control and Vector groups were significantly higher than that in the Sham group (both *p* < 0.001). Importantly, there was no significant difference in the number of TUNEL-positive cells between the Control and Vector groups (*p* > 0.05), indicating that the AAV vector itself did not influence neuronal survival. In contrast, neuronal apoptosis was significantly inhibited in the LncRNA CRNDE group compared to the Control group (*p* < 0.001), demonstrating the neuroprotective effect of CRNDE overexpression.

### 3.6 CRNDE overexpression attenuated histologic damages induced by spinal cord ischemia

**Figure 6:**
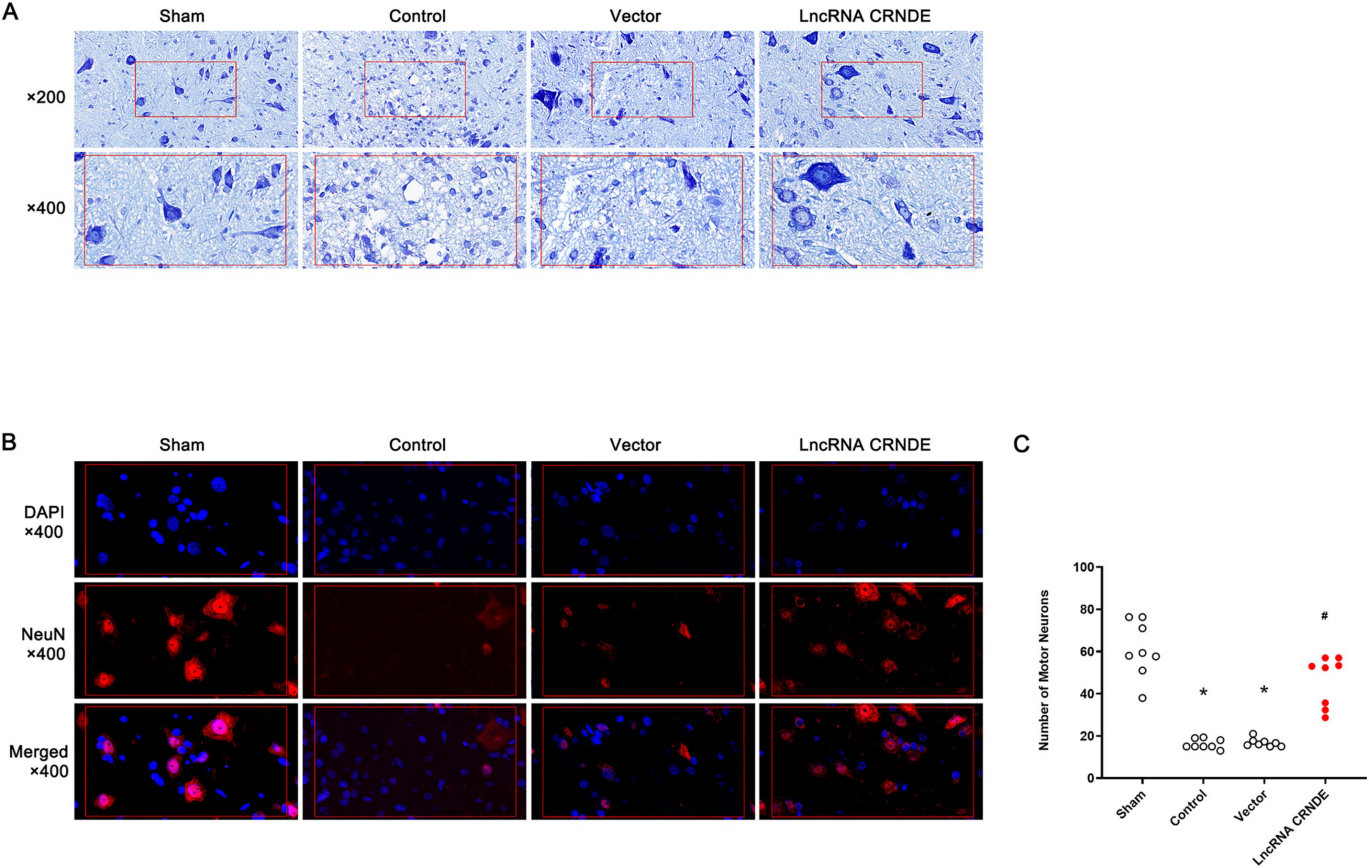
Histologic examinations of lumber spinal cords. **(A)** Images of Nissl staining sections. **(B)** Immunofluorescent images of neurons double stained with NeuN (red) and 4′6-diamidino-2-phenylindole (DAPI) (blue). **(C)** Number of motor neurons in the ventral gray matter of lumber spinal cords. *p < 0.008 versus Sham. ^#^*p* < 0.008 versus Control.

The Nissl staining sections of the Sham group demonstrate normal histologic structure with intact motor neurons, exhibiting a clear shape and abundant cytoplasm (Figure 6A). Conversely, the Control and Vector groups exhibited severe neurological impairment, characterized by vacuolation, frank necrosis, and loss of motor neurons. In contrast, only slight histologic changes were demonstrated in the LncRNA CRNDE group.

As illustrated in Figure 6B, surviving motor neurons in spinal cords marked by NeuN (red fluorescence) are shown. Following spinal cord ischemia, there was a significant decrease in viable motor neurons in both the Control group and Vector group (*p* = 0.001 and *p* = 0.001, respectively, vs. the Sham group) (Figure 6C). The number of surviving motor neurons in the LncRNA CRNDE group was significantly higher than in the Control group (*p* = 0.001).

## 4 Discussion

CRNDE functions as a pivotal regulator of pathological tissue injury, extending its established role as an oncogene. It exerts cardioprotection by alleviating sepsis-induced cardiomyocyte apoptosis and oxidative damage (13). In hepatic models, CRNDE upregulation enhances *in vitro* cell viability and ameliorates sepsis-induced liver injury in rats (19). Furthermore, CRNDE modulates the Yes-associated protein (YAP) signaling pathway to inhibit apoptosis and shield the myocardium against ischemia–reperfusion injury (14, 20). Conversely, CRNDE expression increases time-dependently in the neonatal brain following hypoxic–ischemic insult, where its silencing alleviates damage by promoting autophagy, as opposed to its overexpression (21).

This study characterizes the regulatory mechanism of CRNDE in spinal cord ischemia for the first time. Our data show that CRNDE is downregulated in the spinal cord following transient ischemia, while its overexpression confers significant neuroprotection. This efficacy is evidenced by preserved motor function, suppressed neuronal apoptosis, and increased survival of intact motor neurons. Notably, a recent report on cerebral ischemia identified CRNDE as a target of METTL3-mediated m6A modification, where its inhibition stabilizes ATG10 mRNA to enhance autophagy (22). In contrast, our findings highlight a distinct regulatory dimension in the spinal cord, where CRNDE appears to function through a competitive endogenous RNA mechanism to restore downstream signaling. These observations suggest that the biological role of CRNDE in tissue injury is highly context-dependent, varying across organs and models. Factors such as the tissue-specific microenvironment and the temporal stage of ischemia likely determine the specific expression profile and functional output of CRNDE.

The role of CRNDE as a molecular sponge is well-documented in various malignancies, where it targets microRNAs such as miR-384 in glioma (23) and miR-345-5p in leukemia (24). Building on this framework, we identified miR-181a-5p as a novel target of CRNDE, with direct binding confirmed by dual-luciferase reporter assays. Our *in vivo* data revealed that spinal cord ischemia significantly reduces CRNDE levels while concurrently increasing miR-181a-5p expression; importantly, CRNDE overexpression effectively reversed this ischemic induction. This reciprocal negative regulatory relationship is consistent with the established role of miR-181a-5p as a key regulator of CNS ischemia. Elevated circulating levels of miR-181a-5p have been reported in stroke patients (25), and its inhibition mediates the neuroprotective effects of lncRNA SNHG12 against oxygen–glucose deprivation-induced neuronal apoptosis (26). Furthermore, suppressing miR-181a-5p activates the Wnt/β-catenin signaling pathway by targeting Engrailed-2, which in turn attenuates cerebral ischemic injury (27). By sequestering miR-181a-5p, CRNDE likely neutralizes a conserved mediator of ischemic damage, providing a mechanism for the observed neuroprotection in the spinal cord.

Bioinformatics analysis and dual-luciferase assays confirmed Sirt1 as a direct target of miR-181a-5p. Sirt1, a NAD^+^-dependent deacetylase, serves as a critical neuroprotective factor in CNS ischemia by reducing infarct volume and modulating inflammatory and apoptotic pathways, including p53 and NF-κB (28). For instance, Maresin 1 treatment attenuates cerebral ischemia–reperfusion injury by activating Sirt1 signaling to mitigate mitochondrial damage (29). Similarly, lncRNA SNHG12 has been shown to upregulate Sirt1 by sponging miR-199a, thereby activating the AMPK pathway to alleviate cerebral injury (30). Notably, beyond its established function as a microRNA sponge, CRNDE has recently been shown to directly interact with and stabilize the SIRT1 protein, thereby influencing downstream signaling pathways (31). This suggests that CRNDE may exert its neuroprotective effects through a sophisticated dual regulatory mode, involving both the indirect upregulation of Sirt1 mRNA via miR-181a-5p sequestration and the direct stabilization of the SIRT1 protein. Our study utilized a CRNDE overexpression model to evaluate functional deficits, neuronal apoptosis, and cell loss in the ischemic spinal cord. The results indicate that CRNDE confers robust neuroprotection by downregulating miR-181a-5p, which in turn restores Sirt1 expression and activates its downstream protective signaling.

These downstream targets were selected because FOXO1 and the HIF-1α/PHD2 pathway are established effectors of Sirt1-dependent antioxidant, anti-apoptotic, and hypoxia-adaptive responses in CNS ischemia. FOXO1 is a direct target of Sirt1 deacetylation, which in turn induces its transcriptional activity on the Sirt1 promoter as well as on reactive oxygen species scavenger promoters (32). The Sirt1/FOXO1 pathway plays a vital role in regulating oxidative stress, the inflammatory reaction and apoptosis. The activation of the Sirt1/FOXO1 pathway has been shown to alleviate neuron injury in an oxygen–glucose deprivation model, a process that may be attributed to the antioxidant activity and anti-apoptotic effects of this pathway (33). Furthermore, the activation of the Sirt1/FOXO1 pathway has been shown to mediate the neuroprotective effects of piceatannol (34) and electroacupuncture (35) against cerebral ischemia–reperfusion injury, possibly by suppressing apoptosis, oxidative stress, and autophagy.

Parallel to the FOXO1 axis, HIF-1α acts as a master transcriptional regulator that is rapidly induced by hypoxia through post-transcriptional mechanisms (36). Although its role in cerebral ischemia remains a subject of debate, possibly due to variations in ischemic duration and severity (37), HIF-1α has been shown to attenuate ischemic damage by modulating glial activity and the inflammatory response (38). Sirt1 directly targets HIF-1α to regulate its stability and transcriptional output (39). In hypoxic conditions, Sirt1 inhibition decreases HIF-1α protein accumulation (40), while Sirt1-induced downregulation of the oxygen sensor PHD2 is essential for HIF-1α stabilization (41). Notably, PHD2 knockdown provides protection against hypoxic injury by augmenting HIF-1α (42), while pyruvate has been shown to protect the spinal cord against IRI by inhibiting PHD2 and promoting the HIF-1α/BNIP3 pathway (43). In the present study, the concurrent enhancement of FOXO1 and HIF-1α, coupled with the downregulation of PHD2, was closely associated with Sirt1 restoration. These findings indicate that CRNDE overexpression does not merely target a single pathway; instead, it orchestrates a multi-faceted neuroprotective response. By sequestering miR-181a-5p and restoring Sirt1, CRNDE activates a synergistic network that couples FOXO1-mediated antioxidant defense with HIF-1α-dependent hypoxic adaptation. This crosstalk between Sirt1 and its downstream targets, including FOXO1, PHD2, and HIF-1α, underpins the robust neuroprotection observed in our spinal cord ischemia model, providing a potential therapeutic strategy that addresses the complex pathological milieu of spinal cord injury.

Although CRNDE overexpression demonstrated potent neuroprotection, several limitations should be noted. First, the therapeutic necessity of endogenous CRNDE remains to be fully validated through loss-of-function experiments, such as CRNDE knockdown. Second, while the modulation of FOXO1, HIF-1α, and PHD2 aligns with Sirt1 restoration, we did not perform direct functional inhibition of these specific downstream effectors. Consequently, these proteins should be considered associated mediators of the CRNDE/Sirt1 axis. Future studies incorporating rescue assays and long-term functional assessments will be essential to further dissect the precise contribution of each component within this protective network.

## 5 Conclusion

In conclusion, we identified CRNDE/miR-181a-5p/Sirt1 as a previously unreported ceRNA axis in spinal cord ischemia–reperfusion injury, with downstream involvement of FOXO1, HIF-1α, and PHD2, establishing a novel multi-node neuroprotective pathway with potential clinical relevance for preventing spinal ischemic injury.

## Data Availability

The raw data supporting the conclusions of this article will be made available by the authors, without undue reservation.
