# Supplementary material for: Overexpression of long noncoding RNA colorectal neoplasia differentially expressed protects spinal cords against ischemia by targeting microRNA-181a-5p/Sirtuin-1 axis
Source: Front Neurol. 2026 May 12;17:1825718. doi: 10.3389/fneur.2026.1825718 (PMC13201118; doi:10.3389/fneur.2026.1825718)
Supplement: Supplementary file 1 [file Presentation_1.pptx]

## Slide 1
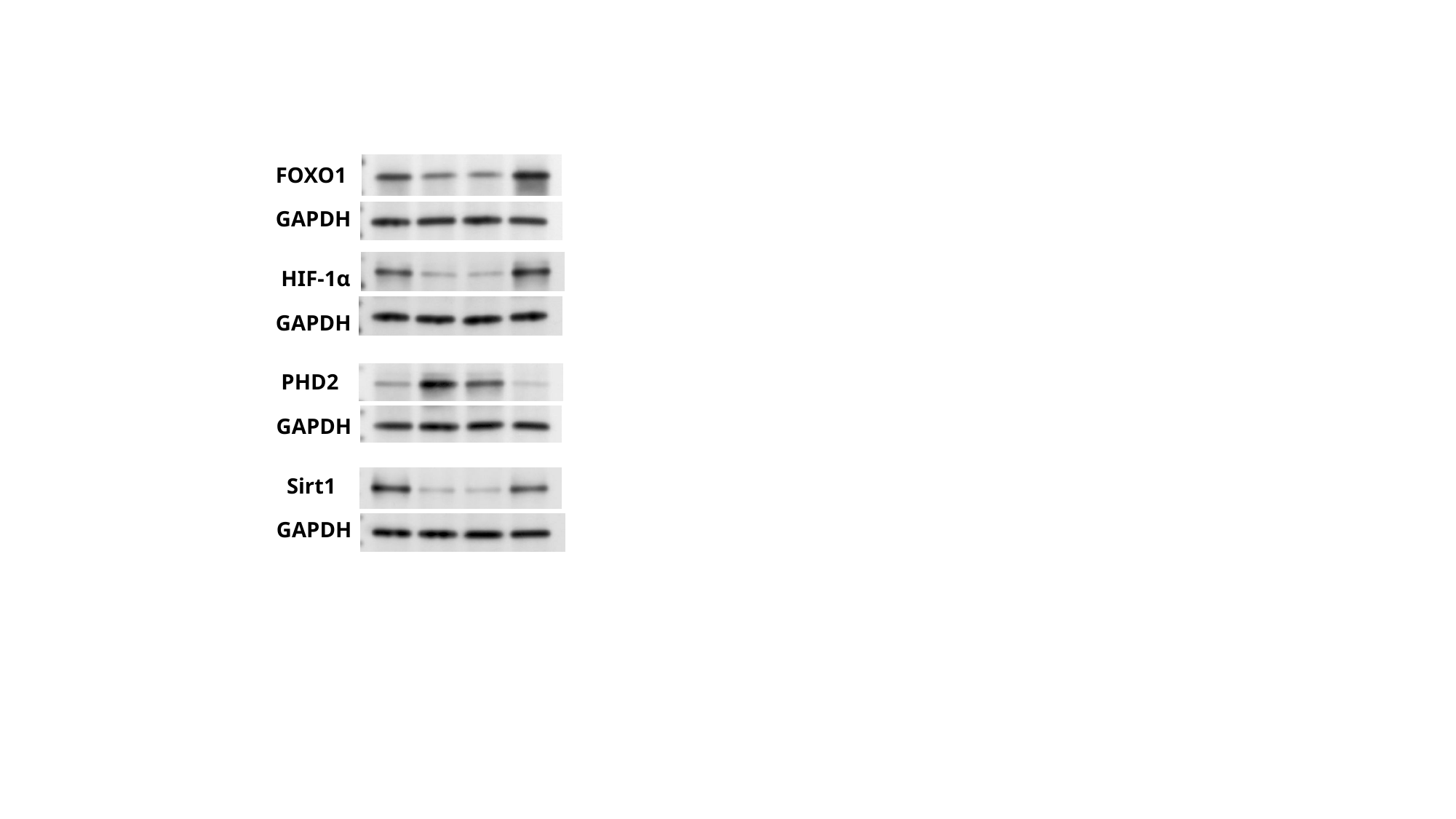

FOXO1
GAPDH
HIF-1α
GAPDH
PHD2
GAPDH
Sirt1
GAPDH

## Slide 2
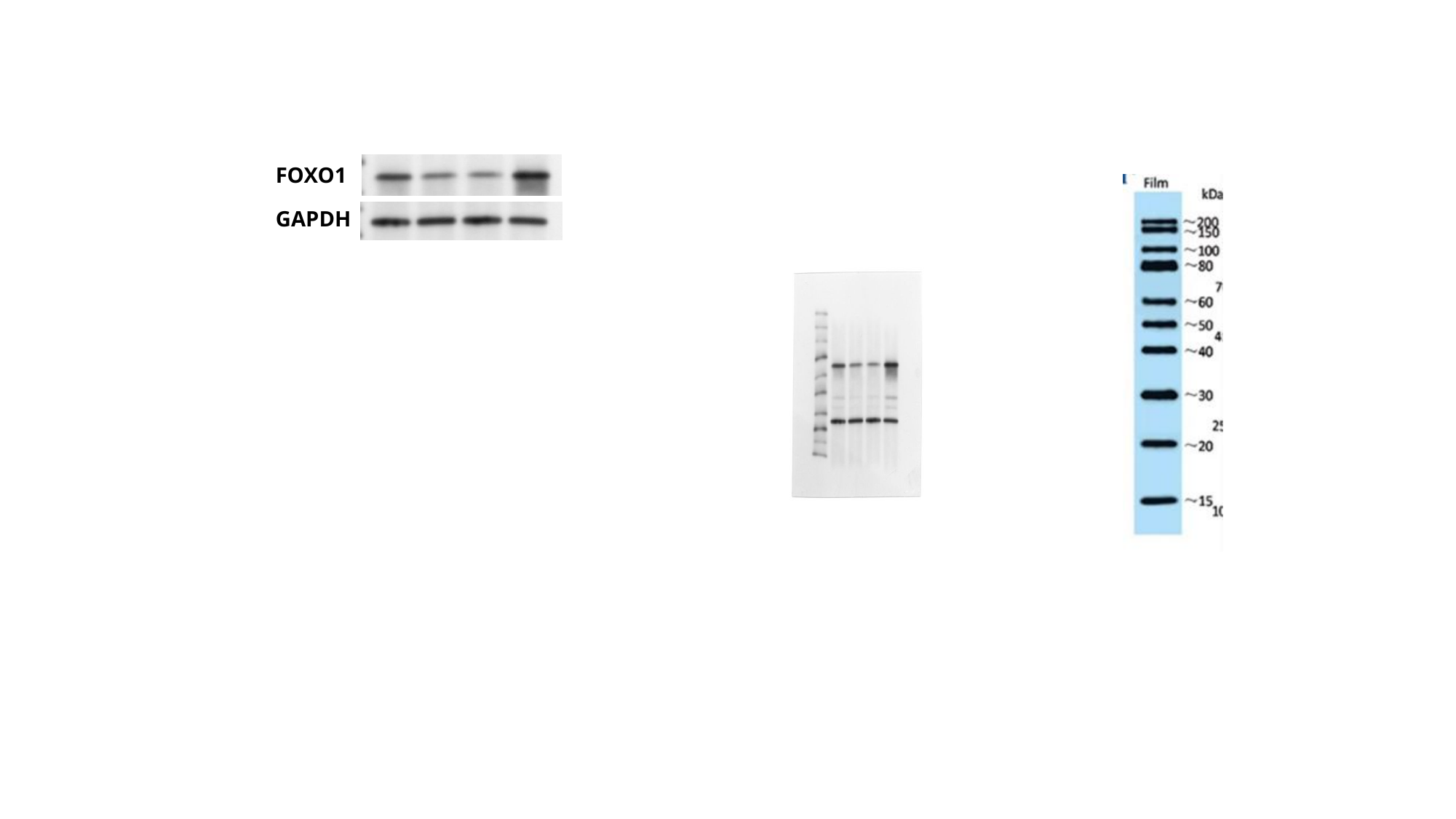

FOXO1
GAPDH

## Slide 3
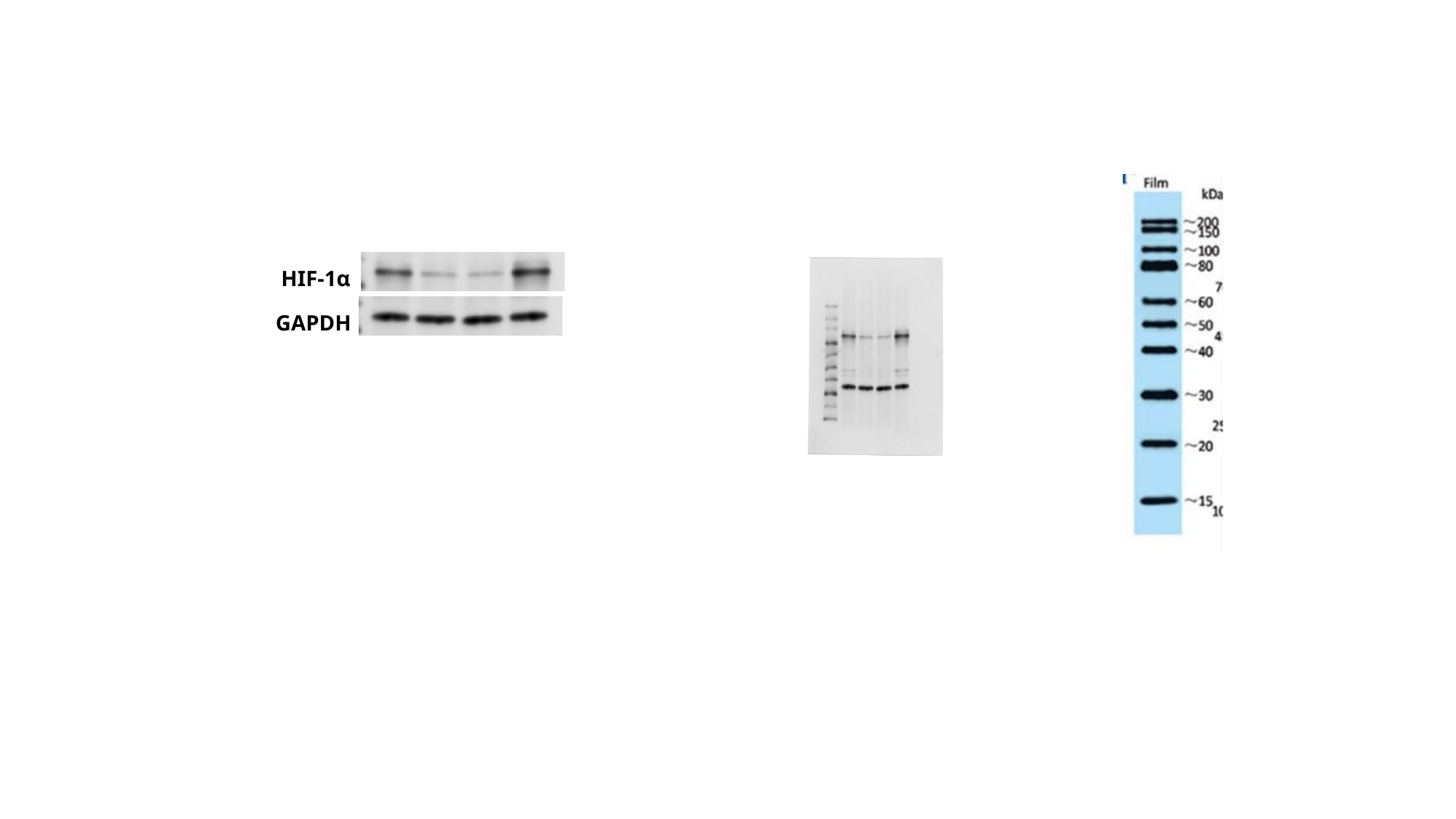

HIF-1α
GAPDH

## Slide 4
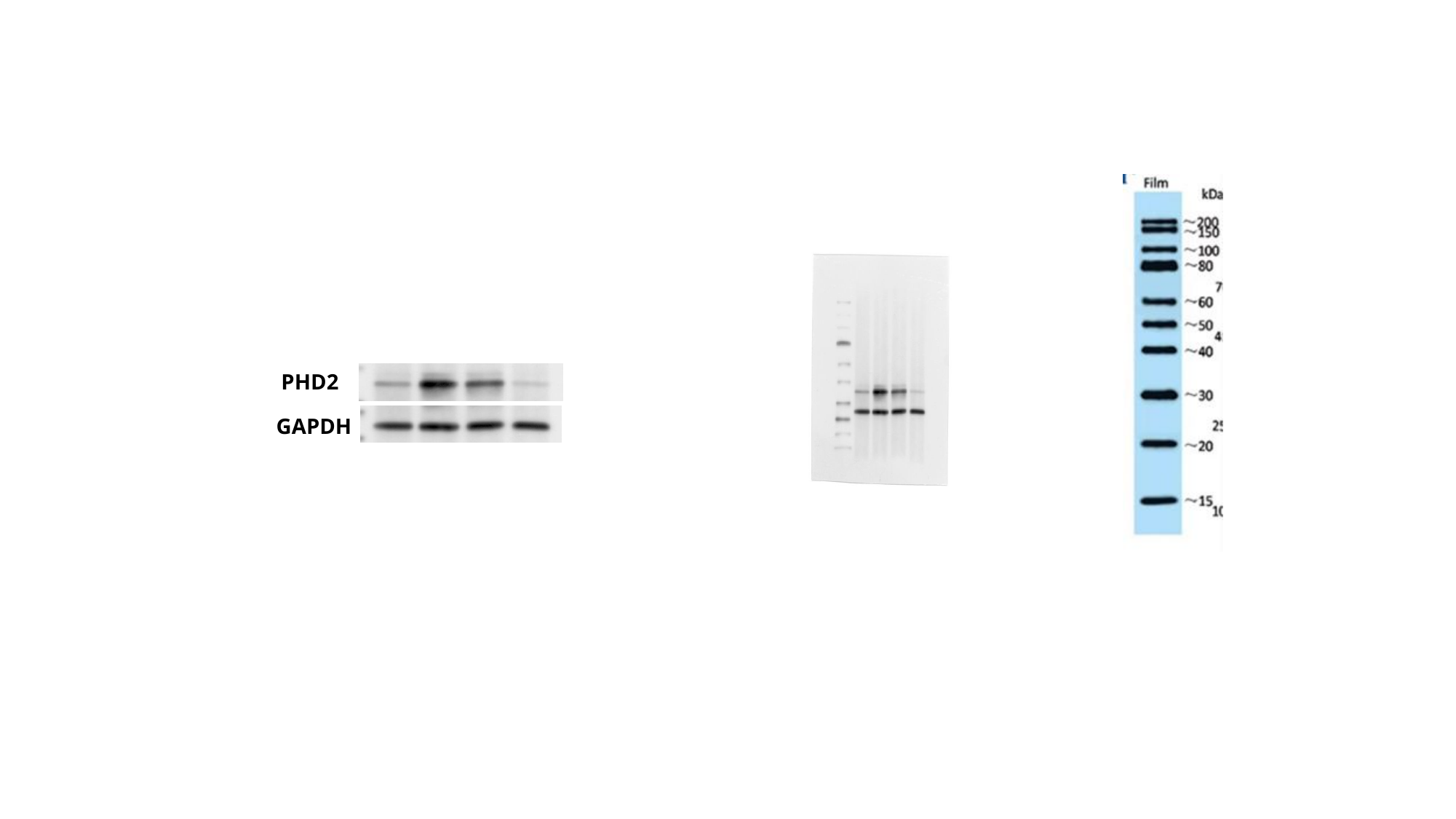

PHD2
GAPDH

## Slide 5
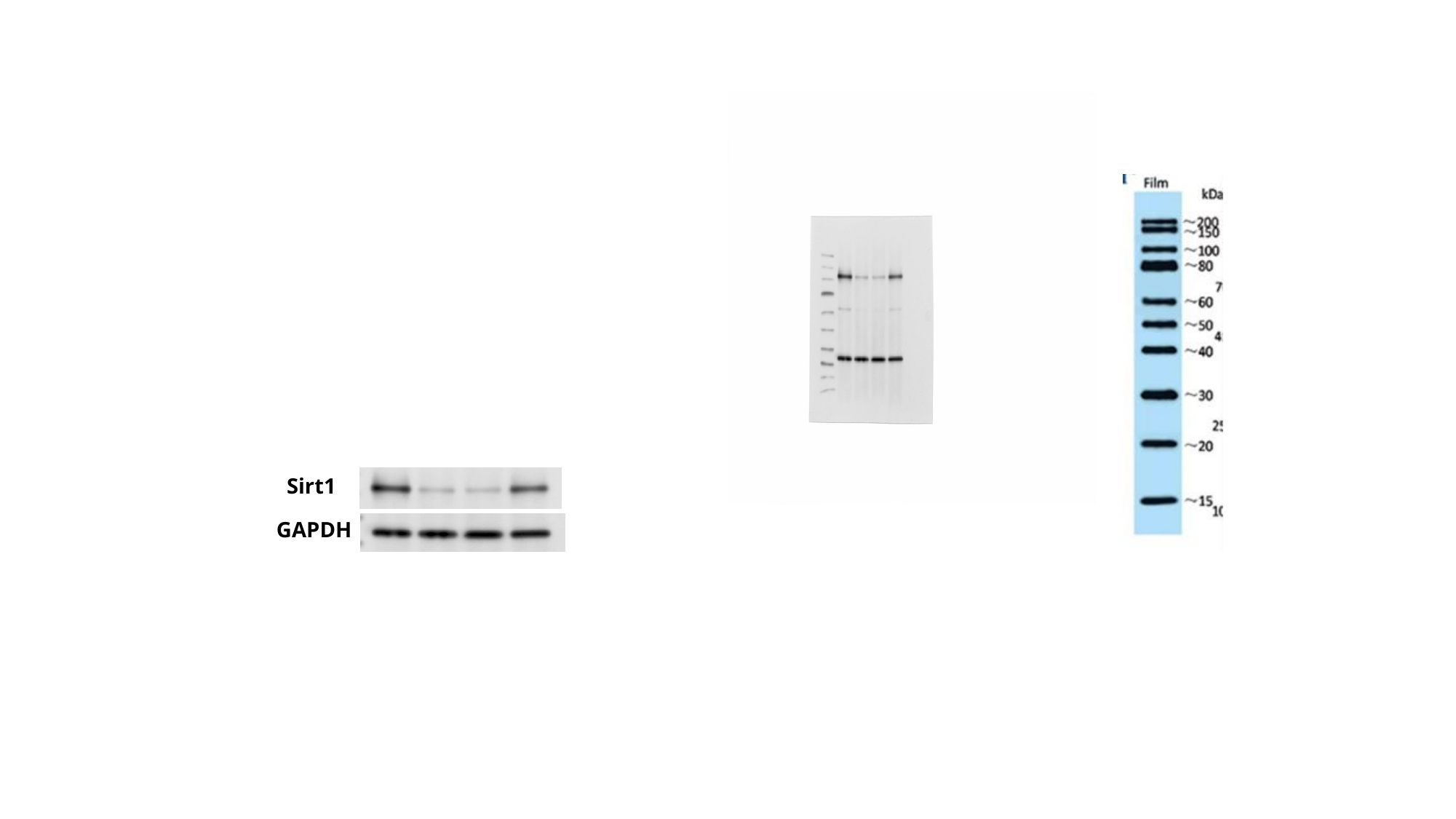

Sirt1
GAPDH
